# Prevalence of significant depressive symptoms and associated factors among facially disfigured pediatric Noma patients in Nigeria: A single-centre cross-sectional study

**DOI:** 10.1371/journal.pmen.0000583

**Published:** 2026-04-17

**Authors:** Olalekan Vincent Ata, Ruth Ifeoluwa Oladele, Ifeoluwa Adetula, Deborah Iyanuoluwa Akinrinde, Kehinde David Arokoyo

**Affiliations:** 1 Faculty of Dentistry, College of Medicine, University of Ibadan, Ibadan, Nigeria; 2 Mental Health Innovation, Leadership and Entrepreneurship Centre (MHILE), Slum and Rural Health Initiative, Ibadan, Nigeria; 3 Department of Management, London School of Economics and Political Science, London, United Kingdom; 4 Zeronoma Initiative, Kano, Nigeria; 5 International Youth Neuroscience Association, Lagos, Nigeria; Santa Casa de Sao Paulo School of Medical Sciences, BRAZIL

## Abstract

Noma is a neglected tropical disease that causes severe facial disfigurement and significant morbidity, yet there is limited evidence regarding the psychological well-being of affected children. This study aimed to determine the prevalence of depressive symptoms and identify associated factors among pediatric Noma patients attending the Noma Children Hospital in Sokoto, Nigeria. A cross-sectional study was conducted involving 244 pediatric patients aged 6–16 years who had been facially disfigured by Noma and were undergoing rehabilitation or awaiting surgery. Data were collected using a structured questionnaire that included sociodemographic characteristics and the Center for Epidemiologic Studies Depression Scale (CES-D) to assess depressive symptoms. Binary logistic regression was performed to identify predictors of high depressive symptoms. Statistical significance was set at *p* < 0.05. The prevalence of clinically significant depressive symptoms among the participants was 76.6% (95% CI: 70.9-81.4%). Multivariate analysis revealed that gender, parental employment, and household income were associated with depressive symptoms. Female patients were significantly more likely to exhibit high depressive symptoms compared to males (OR = 3.251, 95% CI: 1.499-7.048, p = 0.003). Higher household income was associated with increased odds of depressive symptoms (OR = 3.411, 95% CI: 1.624-7.167, p = 0.001), whereas parental employment was significantly associated with reduced odds (OR = 0.401, 95% CI: 0.217-0.739, p = 0.003). There is a substantial burden of depressive symptoms among pediatric Noma patients in Nigeria, with female gender and family socioeconomic factors significantly shaping the risk. These findings highlight the critical need to integrate mental health screening and psychosocial interventions into Noma care protocols.

## Introduction

Noma, also known as cancrum oris, is a devastating infectious disease that primarily affects the face, leading to severe facial disfigurement and significant morbidity [1]. The disease begins as a small gingival ulcer and rapidly extends to involve facial tissues, leading to massive disfigurement, functional impairment, and high mortality rates if untreated [2]. Although Noma is preventable, it remains a neglected tropical disease associated with severe physical, psychological, and social consequences among survivors [3]. This condition predominantly impacts children living in extreme poverty within tropical regions, where malnutrition, poor sanitation, and limited access to quality healthcare persist [4].

According to the World Health Organization, about 770,000 persons live with Noma [4]. Due to the global health burden and the high mortality and morbidity rate posed by Noma, the World Health Organization (WHO) has recently classified Noma as part of the Neglected Tropical Diseases (NTDs) [5]. In Nigeria, the epidemiology of Noma is particularly noteworthy, with a systematic review by Eleje et al. [6] which estimated that approximately 3 in 100 children in Nigeria had Noma. The age of onset is usually between two to seven years [7].

Beyond its physical consequences, patients with Noma are prone to suffering from mental health conditions due to factors such as lack of social support and low self-esteem, among others [5]. Young people are at an increased risk of having mental disorders with 50% of mental disorders occurring before age 14 and 75% of mental disorders occurring before age 24 [8]. This prevalence of mental disorders among young people under 16 is worsened in children who have an existing medical condition [8]. Studies among children with other chronic disfiguring conditions, such as cleft lip and facial palsy, have reported significant levels of depressive symptoms, highlighting the mental health toll of visible disfigurement [9,10].

Existing literature has assessed the prevalence of depression among adults with Noma, revealing significant psychological distress and a high incidence of depressive disorders [11,12]. Also, several studies have addressed the epidemiology, clinical management, and surgical rehabilitation of pediatric Noma patients [13,14]. However, there is limited evidence on the psychological well-being of affected children. Mental health services are rarely integrated into Noma care, and the emotional needs of survivors often go unrecognized [12].

Given the psychosocial consequences of Noma and the lack of data on mental health outcomes in affected children despite the unique challenges and vulnerabilities of this group, there is a critical need to assess the prevalence and determinants of depressive symptoms in this vulnerable population. Understanding these factors will inform the integration of mental health interventions into Noma care, ensuring a more comprehensive and patient-centered approach to treatment and rehabilitation. This study, therefore, aimed to determine the prevalence of depressive symptoms and identify associated factors among pediatric Noma patients attending the Noma Children Hospital, Sokoto, Nigeria. Findings from this study are expected to provide baseline data to support the integration of mental health screening and support services into Noma care in Nigeria and similar settings.

## Materials and methods

### Ethical statement

Ethical approval was obtained from the Sokoto State Ministry of Health Research Ethics Committee (Approval No: SKHREC/009/2025). Permission to conduct the study was granted by the management of Noma Children Hospital, Sokoto, Nigeria. Informed verbal consent was obtained from parents or guardians, while assent was obtained from all participating children all documented in the individual questionnaire on Kobocollect. Participation was voluntary, and data confidentiality was maintained throughout the study.

Participants who screened positive for severe depressive symptoms (scores ≥16) were referred to the counselling unit and the managing clinical team for further psychological evaluation and support.

### Study design

This is a cross-sectional study aimed at assessing the prevalence of depression in pediatric Noma patients.

### Study site

The study was conducted at the Noma Children’s Hospital, Sokoto, a specialized 60-bed tertiary care facility situated in Northwestern Nigeria. As the primary referral centre for Noma in the country and the surrounding West African sub-region, the hospital serves a vast catchment area of the disease. The centre provides comprehensive, holistic management for patients, integrating reconstructive surgery, nutritional rehabilitation and physiotherapy services. It operates in partnership with the Ministry of Health and international non-governmental organizations (NGOs) to deliver free, high-quality care to this vulnerable population [15].

Patient volume at the centre fluctuates significantly due to quarterly free surgical missions offered by Médecins Sans Frontières, an NGO, in the hospital [16]. This mission draws Noma patients from the regions across the country, increasing the usual caseload. This distinct operational pattern enabled the recruitment of a robust sample size for this study.

### Participants and eligibility criteria

Participants were pediatric patients aged 6–16 years who had been facially disfigured by Noma and were either awaiting surgery or undergoing rehabilitation. Children in critical health conditions requiring immediate medical attention and those with diagnosed neurological disorders were excluded. Participants were required to understand either English or Hausa.

Children aged 6–16 years were chosen for this study because they can understand and respond to questions about their feelings and experiences. Most tools used to assess depression in children are also designed and validated for this age range. Focusing on this group helped to obtain more accurate responses and reduced the need to rely on parents or caregivers to report how the child feels.

### Sample size determination

The sample size was determined using the Kish Leslie formula. Based on a reference prevalence of 17.5% for depressive symptoms [17], a 95% confidence level (Z = 1.96), and a 5% margin of error (d = 0.05), the minimum sample size was calculated as:


$$\text{n }=\;(\text{1}.{\text{96}}^{\text{2}}\;\times \;\text{0}.\text{175 }\times \;\text{0}.\text{825})/\;\text{0}.\text{0025 }=\text{ 221}.\text{8},\text{ approximated to 222}.$$

### Sampling procedure

A non-probability consecutive sampling technique was employed for this study. All pediatric patients presenting with Noma to the hospital during the study were approached for recruitment. This method was selected to minimize selection bias and maximize the sample size, given the relatively low prevalence of the condition.

The minimum sample size was calculated to be 222 participants. However, to maximize the statistical power of the study and account for the unique opportunity presented by the mass intervention campaign ongoing in the hospital during the study period, a total of 254 participants were recruited. All participants met the inclusion criteria.

### Data collection tools

Data were obtained through a structured interviewer-administered questionnaire developed in English and translated into Hausa. The instrument consisted of two main sections:

(1) **sociodemographic characteristics**: Data collected included the child’s age and gender, parental education level, parental employment status (including job type where applicable), household income level, family structure (whether nuclear, extended or single parent family), and the number of children in the household.
(2) **Center for Epidemiologic Studies Depression Scale (CES-D)**: The CES-D is a 20-item self-report tool designed to measure depressive symptomatology in the general population. Each item is rated on a 4-point Likert scale ranging from 0 (“rarely or none of the time”) to 3 (“most or all of the time”), with total scores ranging from 0 to 60. Higher scores indicate more severe depressive symptoms, with a cutoff score of ≥16 suggestive of clinically significant depression. The CES-D has been validated for use in African populations and is suitable for ages 6 years and above [18]. It shows good internal consistency, with Cronbach’s alpha coefficients typically ranging from 0.60 to 0.78, and good test–retest reliability (r = 0.55 over 2 weeks) [19].

To ensure cultural and linguistic appropriateness, we followed the standard forward-backward translation protocol. The instrument was translated from English to Hausa by a bilingual healthcare provider and subsequently back-translated by an independent linguist to ensure equivalence.

Following this, a pilot test was conducted to assess the comprehension of the items among the study population with Cronbach’s alpha score of 0.77.

### Procedure

Research assistants received a two-day structured training on study objectives, questionnaire administration, child safeguarding, and ethical conduct when working with minors. To ensure consistency, role-play sessions were conducted. The principal investigator also conducted random spot-checks during data collection to ensure quality data collection.

Participants and their caregivers were briefed about the purpose and procedures of the study. Data was collected by the researchers and conducted in private, quiet areas within the hospital to ensure confidentiality and comfort. Responses were provided by the participants and recorded using the KoboCollect mobile data-entry platform to enhance data accuracy and minimize loss. Data collection took place between 1^st^ April 2025 and 31^st^ August 2025,

### Statistical analysis

Quantitative data were analyzed using IBM SPSS Statistics version 27. Descriptive statistics including frequencies, percentages, means, and standard deviations were used to summarize sociodemographic characteristics and CES-D scores. Associations between categorical variables were examined using the Chi-square test, while binary logistic regression analysis was performed to identify sociodemographic and family-related predictors of depressive symptoms. Statistical significance was set at *p* < 0.05.

## Result

### Participant flow and sample size

A total of 254 pediatric Noma patients were screened for eligibility, and 244 participants who met the inclusion criteria were included in the data collection.

### Sociodemographic characteristics

The mean age of study participants was 10.93 ± 3.17 years. The proportion of female participants (56.6%) was slightly higher than that of males (43.4%). Socio-demographic characteristics of participants is presented in Table 1.

**Table 1:** Sociodemographic characteristics of study participants (N = 244).

Variable	n	%
**Sex**		
Male	106	43.4
Female	138	56.6
**Parental education**		
None	129	52.9
Primary	48	19.7
Secondary	50	20.5
Tertiary	17	7.0
**Parental employment**		
Civil servant	55	22.5
Not employed	71	29.1
Self-employed	117	48.0
**Monthly income**		
< ₦50,000 (<$34)	127	52.0
₦50,000–₦100,000 ($34-$68)	70	28.7
₦100,001–₦200,000 ($68-$136)	37	15.2
> ₦200,000 (>$136)	10	4.1
**Family structure**		
Extended	54	22.1
Nuclear	81	33.2
Single parent	109	44.7

### Distribution and prevalence of depressive symptoms

Based on the CES-D cut-off score of 16, 187 participants (76.6%) were classified as having clinically significant depressive symptoms, while 57 participants (23.4%) had scores below the threshold, indicating no significant depressive symptoms. This corresponds to a prevalence of 76.6% (95% CI ≈ 70.9–81.4%) for clinically significant depressive symptoms among the study population. The gender and age distribution of the depressive symptoms is represented in Table 2.

**Table 2:** Gender and age distribution of depressive symptoms.

Variable	Significant Depressive symptoms (%)	Low Depressive symptoms (%)
**Sex**		
Male	72 (67.9)	34 (32.1)
Female	115 (83.3)	23 (16.7)
**Age (year)**		
6-9	46 (74.2)	16 (25.8)
10-12	62 (72.1)	24 (27.9)
13-16	64 (80.0)	32 (20.0)

### Bivariate analyses of factors associated with depression

The relationships between depressive symptoms and sociodemographic variables were examined using chi-square tests (Table 3). Gender was significantly related to depression category (χ² = 7.95, p = 0.005), with a higher proportion of females (83.3%) than males (67.9%) exhibiting high depressive symptoms. Parental education level also showed a strong association (χ² = 29.52, p < 0.001), with depressive symptoms more common among children of parents with tertiary (94.1%) and no formal education (84.5%). Household income (χ² = 8.93, p = 0.030) and family structure (χ² = 8.63, p = 0.013) were similarly associated with depressive symptoms, while no significant relationships were found with parental employment status (χ² = 5.07, p = 0.167), age of child (χ² = 30.08, p = 0.018; interpreted with caution due to low expected counts), or number of children in the family (χ² = 17.82, p = 0.164).

**Table 3:** Bivariate associations between socio-demographic characteristics and depressive symptoms among pediatric Noma patients.

Variable	χ²	df	p-value
Gender	7.951	1	0.005
Parental Education	29.519	3	<0.001
Family Structure	8.632	2	0.013
Household Income	8.926	3	0.030
Parental Employment	5.071	3	0.167

### Multivariate Analyses of Factors Associated with Depression

A binary logistic regression was conducted to examine the factors associated with high depressive symptoms. Variables were included in the multivariable model if they demonstrated a statistically significant association (p < 0.05) in the bivariate analysis (Gender, Parental Education, Household Income and Family Structure) or were deemed theoretically relevant based on existing literature (Age of Child and Parental Employment). Listwise deletion was employed for handling missing data. Only participants with complete data on all covariates were included in the final model (N = 183).

Significant associated factors included gender, parental employment status, and household income as shown in Table 4.

**Table 4:** Logistic Regression of Factors Associated with High Depressive Symptoms (N = 183).

Predictor	p-value	OR	95% CI for OR
Age of child	.093	0.892	0.780 – 1.019
Gender of child (female)	.003*	3.251	1.499 – 7.048
Parental education	.447	0.815	0.480 – 1.382
Parental employment	.003*	0.401	0.217 – 0.739
Household income	.001*	3.411	1.624 – 7.167
Family structure	.093	0.620	0.355 – 1.083
Constant	.054	6.862	—

MODEL FIT:

• χ² (7, N = 183) = 33.33, p < .001.

• Cox & Snell R² = 0.167.

• Nagelkerke R² = 0.244.

• Classification accuracy = 76%.

OR = Odds Ratio.

* = Statistically significant.

## Discussion

This study revealed a high prevalence of depressive symptoms (76.6%) among pediatric Noma patients in Northwestern Nigeria. This rate far exceeds estimates from community-based studies of depressive symptoms in general pediatric populations globally and across Nigeria with reported prevalence ranging between 0.4-12% [20,21]. Ogunlade et al. [22] conducted a meta-analysis that revealed a prevalence of 12% of major depressive symptoms among children and adolescents aged <18 years in Nigeria from 2010 to 2024. The findings emphasize the deep psychosocial burden associated with Noma, a condition marked by disfiguring facial lesions, social stigma, and functional impairment.

The multivariate regression analysis identified a specific high-risk profile, isolating the most direct associated factors of depressive symptoms. The most significant associated factor was gender with female patients 3.25 times more likely to exhibit high depressive symptoms than their male counterparts. This finding is similar to a meta-analysis of population-based surveys conducted by Salk et al. [23] which reported that more adolescent girls than boys had depressive symptoms/depression. In the context of Noma, this disparity may be tragically amplified, probably because of the facial disfigurement that is characteristic of Noma. Globally, societal pressures regarding physical appearance are often more intensely focused on girls, with adolescent girls more likely to be body-shamed compared to their male counterparts [24]. Hence, it is plausible that the characteristic facial disfigurement caused by Noma could be responsible for psychological trauma, internalized stigma, and social rejection in female patients, making the prevalence of depression/depressive symptoms higher in this population group.

Studies on mental health conditions with an attendant facial disfigurement/weakness have reported similar on the higher prevalence of depression among female adolescents and adults. Richards et al. [25] reported a gender difference in the reported level of depression among participants with ptosis. Almost half of the females of the participants report depression as opposed to 20% of the male participants.

Inverse association was observed with parental employment (OR = 0.401, 95% CI: 0.217-0.739, p = 0.003). Parental employment was associated with a 60% reduction in the odds of clinically significant depressive symptoms. Strikingly, however, higher household income emerged as an associated factor (OR = 3.411, 95% CI: 1.624-7.167, p = 0.001). While these findings appear paradoxical, they likely reflect the complex socioeconomic landscape of Nigeria. In the Nigerian context, employment does not invariably translate to financial security. With approximately 93% of the workforce engaged in the informal sector, the enforcement of the national minimum wage (₦77,000 = $52) is largely limited to the strictly regulated public and private sectors. Consequently, for the majority, parental employment may represent subsistence-level activity rather than wealth accumulation.

Conversely, the elevated depression risk among higher-income households (earning > ₦200,000 equivalent of $136) may be mediated by psychosocial factors rather than economic ones. Since Noma is frequently stigmatized as a ‘disease of extreme poverty,’ wealthier families may experience heightened status anxiety or shame. This could compel families to conceal the affected child to protect their social standing, inadvertently leading to greater social isolation and loneliness for the child.

It is equally important to note which factors were not significant in the final regression model. Both parental education and family structure, which were significant in the initial bivariate analysis, lost their predictive power when income and employment were taken into account. This strongly suggests that the effects of education and family structure are not direct but are instead mediated or explained by the more proximal factors of household income and employment status. For instance, the effect of parental education is likely captured within the more dominant influence of income. The regression model successfully “unmasked” the true, associated factors, demonstrating that financial stability (employment) and the complex social dynamics of income are more directly tied to the child’s mental health than family structure or education level alone.

### Policy and program recommendations

The findings from this study have urgent and clear implications. The high prevalence of depressive symptoms suggests that mental health screening must be a core component of all Noma treatment protocols. Psychosocial support should be integrated into care from the moment of diagnosis. Further research can be conducted to assess the feasibility of integrating mental health and psychosocial support services into the regular care of pediatric Noma patients.

Furthermore, interventions should be targeted. Female patients represent a high-risk group and should be prioritized for psychological evaluation and support. Future research should, studies are needed to evaluate the impact of societal awareness campaigns. Finally, supporting the family unit, particularly through interventions that promote parental employment and financial stability is important. This may be one of the most effective long-term strategies for protecting the mental health of these vulnerable children.

### Strengths and limitations

The primary strength of this study lies in its robust sample size, particularly given the rarity of Noma as a neglected tropical disease. Also, conducting the study at a dedicated high-volume tertiary center and at a period of mass intervention campaign allowed for the recruitment of a geographically diverse cohort, enhancing the representativeness of the findings. This study focused on a highly vulnerable population, providing important mental health data on an under-researched group. The use of the standardized and validated CES-D screening tool ensures that the findings are objective, credible, and comparable to other depression studies.

The reliance on the CES-D rather than clinical interviews could have inflated prevalence estimates, which could have influenced the study’s findings. Finally, as this study recruited patients undergoing rehabilitation for visible facial disfigurement, the sample reflects survivors with severe sequelae. This focus on severe cases likely contributes to the high prevalence of depressive symptoms observed.

## Conclusion

The study highlighted the alarmingly high prevalence of depressive symptoms among pediatric Noma patients in Northwestern Nigeria. It also identified socio-demographic factors specifically gender, parental employment, and household income that influence the psychological well-being of these survivors. Effective care models and rehabilitation programs should be evidence-based and holistic. It must include the integration of mental health screening and support services. This comprehensive approach will help ensure that affected children have access to the patient-centered care they need for full recovery, thereby mitigating the severe psychosocial consequences of the disease.

Complex socioeconomic dynamics, such as the paradoxical relationship between higher household income and increased depression risk potentially driven by stigma and status anxiety, were identified as significant influences and are recommended for areas of further research.

## References

[1] Yunusa M, Obembe A. Prevalence of psychiatric morbidity and its associated factors among patients facially disfigured by cancrum oris in Nigeria a controlled study. Niger J Med. 2012;21(3):277–81. 23304920

[2] Baratti-Mayer D, Pittet B, Montandon D, Bolivar I, Bornand J-E, Hugonnet S, et al. Noma: an “infectious” disease of unknown aetiology. Lancet Infect Dis. 2003;3(7):419–31. doi: 10.1016/s1473-3099(03)00670-4 12837347

[3] Srour ML, Baratti-Mayer D. Why is noma a neglected-neglected tropical disease?. PLoS Negl Trop Dis. 2020;14(8):e0008435. doi: 10.1371/journal.pntd.0008435 32817617 PMC7444552

[4] World Health Organization Regional Office for Africa. Information brochure for early detection and management of noma [Internet]. 2017. Available from: https://apps.who.int/iris/handle/10665/254579

[5] World Health Organization. WHO officially recognizes noma as a neglected tropical disease [Internet]. 2023 Dec 15 [cited 2025 Dec 7]. Available from: https://www.who.int/news/item/15-12-2023-who-officially-recognizes-noma-as-a-neglected-tropical-disease

[6] Eleje GU, Okoh EE, Igbodike EP, Akinsolu FT, Nwaokorie FO, Lusher JM, et al. Prevalence and associated risk factors for noma in Nigerian children: a systematic review and meta-analysis. BMC Oral Health. 2024;24(1):685. doi: 10.1186/s12903-024-04451-y 38867180 PMC11170919

[7] Srour ML, Marck K, Baratti-Mayer D. Noma: Overview of a Neglected Disease and Human Rights Violation. Am J Trop Med Hyg. 2017;96(2):268–74. doi: 10.4269/ajtmh.16-0718 28093536 PMC5303022

[8] Versnel SL, Plomp RG, Passchier J, Duivenvoorden HJ, Mathijssen IMJ. Long-term psychological functioning of adults with severe congenital facial disfigurement. Plast Reconstr Surg. 2012;129(1):110–7. doi: 10.1097/PRS.0b013e3182361f64 21915083

[9] Fu L, Bundy C, Sadiq SA. Psychological distress in people with disfigurement from facial palsy. Eye (Lond). 2011;25(10):1322–6. doi: 10.1038/eye.2011.158 21720412 PMC3194312

[10] Braimah RO, Bakare AT, Yakubu AI, Taiwo AO, Bala M, Fatusi OA, et al. Psychiatric morbidities and quality of life among surgically treated noma survivors. Preliminary observations in Nigerian cohorts. J Stomatol Oral Maxillofac Surg. 2025;126(4S):102315. doi: 10.1016/j.jormas.2025.102315 40090548

[11] Gebretsadik HG. The severity of psychosocial and functional morbidity among facially disfigured untreated noma cases in Ethiopia. BMC Res Notes. 2023;16(1):162. doi: 10.1186/s13104-023-06440-w 37550768 PMC10408114

[12] Onu JU, Aluh DO, Ononiwu CN. Psychosocial aspects of Noma (Cancrum Oris) in sub-Saharan Africa: A scoping review. Trop Doct. 2023;53(4):470–4. doi: 10.1177/00494755231175529 37165663

[13] Farley E, Mehta U, Srour ML, Lenglet A. Noma (cancrum oris): A scoping literature review of a neglected disease (1843 to 2021). PLoS Negl Trop Dis. 2021;15(12):e0009844. doi: 10.1371/journal.pntd.0009844 34905547 PMC8670680

[14] Maguire BJ, Shrestha R, Dahal P, Ngu R, Nizigama L, Rashan S, et al. A systematic review of the noma evidence landscape: Current knowledge and gaps. bioRxiv. 2025. doi: 10.1101/2025.02.07.24315593 PMC12315006 40744672

[15] Isah S, Amirtharajah M, Farley E, Semiyu Adetunji A, Samuel J, Oluyide B, et al. Model of care, Noma Children’s Hospital, northwest Nigeria. Trop Med Int Health. 2021;26(9):1088–97. doi: 10.1111/tmi.13630 34080264 PMC9292046

[16] Sokoto Noma Hospital – Noma [Internet]. Médecins Sans Frontières; [cited 2025 Dec 7]. Available from: https://noma.msf.org/sokoto-noma-hospital

[17] Molla GL, Sebhat HM, Hussen ZN, Mekonen AB, Mersha WF, Yimer TM. Depression among Ethiopian Adults: Cross-Sectional Study. Psychiatry J. 2016;2016:1468120. doi: 10.1155/2016/1468120 27247932 PMC4876238

[18] Getnet B, Alem A. Validity of the Center for Epidemiologic Studies Depression Scale (CES-D) in Eritrean refugees living in Ethiopia. BMJ Open. 2019;9(5):e026129. doi: 10.1136/bmjopen-2018-026129 31064806 PMC6528005

[19] Tatar A, Kayiran SM, Saltukoglu G, Ozkut EŞZ, Emeksiz M. Analysis of the Center for Epidemiologic Studies Depression Scale (CES-D) in Children and Adolescents from the Perspective of the Item Response Theory. Bull Clin Psychopharmacol. 2013;23(3):242–53.

[20] Lima LS, Ribeiro GS, Aquino SN de, Volpe FM, Martelli DRB, Swerts MSO, et al. Prevalence of depressive symptoms in patients with cleft lip and palate. Braz J Otorhinolaryngol. 2015;81(2):177–83. doi: 10.1016/j.bjorl.2015.01.004 25716190 PMC9449002

[21] DadeMatthews A, Nzeakah C, Onofa L, DadeMatthews O, Ogundare T. Teenage Blues: Predictors of depression among adolescents in Nigeria. PLoS One. 2024;19(4):e0293995. doi: 10.1371/journal.pone.0293995 38630744 PMC11023510

[22] Ogunlade SB, Abioye AI, Kuyebi MA, Musa J, Abubakar AK, Yisa MN, et al. Prevalence of psychiatric disorders among children and adolescents in Nigeria since 2010: A systematic review and meta-analysis. bioRxiv [Preprint]. 2025 Feb 22. Available from: doi: 10.1101/2025.02.22.25322718

[23] Salk RH, Hyde JS, Abramson LY. Gender differences in depression in representative national samples: Meta-analyses of diagnoses and symptoms. Psychol Bull. 2017;143(8):783–822. doi: 10.1037/bul0000102 28447828 PMC5532074

[24] Merino M, Tornero-Aguilera JF, Rubio-Zarapuz A, Villanueva-Tobaldo CV, Martín-Rodríguez A, Clemente-Suárez VJ. Body Perceptions and Psychological Well-Being: A Review of the Impact of Social Media and Physical Measurements on Self-Esteem and Mental Health with a Focus on Body Image Satisfaction and Its Relationship with Cultural and Gender Factors. Healthcare (Basel). 2024;12(14):1396. doi: 10.3390/healthcare12141396 39057539 PMC11276240

[25] Richards HS, Jenkinson E, Rumsey N, White P, Garrott H, Herbert H, et al. The psychological well-being and appearance concerns of patients presenting with ptosis. Eye (Lond). 2014;28(3):296–302. doi: 10.1038/eye.2013.264 24357840 PMC3965808

